# A primary luminal/HER2 negative breast cancer patient with mismatch repair deficiency

**DOI:** 10.1038/s41420-023-01650-4

**Published:** 2023-10-02

**Authors:** Xue Yang, Artem Smirnov, Oreste Claudio Buonomo, Alessandro Mauriello, Yufang Shi, Julia Bischof, Jonathan Woodsmith, TOR CENTRE, Pierluigi Bove, Valentina Rovella, Manuel Scimeca, Giuseppe Sica, Giuseppe Tisone, Ying Wang, Francesca Servadei, Gerry Melino, Eleonora Candi, Francesca Bernassola

**Affiliations:** 1 Department of Experimental Medicine, TOR, University of Rome Tor Vergata, 00133 Rome, Italy; 2 The Third Affiliated Hospital of Soochow University, Institutes for Translational Medicine, Soochow University, Suzhou, 215000 China; 3 Istituto Dermopatico Immacolata (IDI-IRCCS), 00100 Rome, Italy; 4 Indivumed GmbH, Falkenried, Germany Biochemistry Laboratory, 88 Building D, 20251 Hamburg, Germany; 5 Deutsches Zentrum für Neurodegenerative Erkrankungen (DZNE), Bonn, Germany; 6 Shanghai Institute of Nutrition and Health, Shanghai, 200031 China

**Keywords:** Breast cancer, Diagnostic markers

## Abstract

Here, we present the case of a 47-year-old woman diagnosed with luminal B breast cancer subtype and provide an in-depth analysis of her gene mutations, chromosomal alterations, mRNA and protein expression changes. We found a point mutation in the *FGFR2* gene, which is potentially hyper-activating the receptor function, along with over-expression of its ligand *FGF20* due to genomic amplification. The patient also harbors somatic and germline mutations in some mismatch repair (MMR) genes, with a strong MMR mutational signature. The patient displays high microsatellite instability (MSI) and tumor mutational burden (TMB) status and increased levels of CTLA-4 and PD-1 expression. Altogether, these data strongly implicate that aberrant FGFR signaling, and defective MMR system might be involved in the development of this breast tumor. In addition, high MSI and TMB in the context of CTLA-4 and PD-L1 positivity, suggest the potential benefit of immune checkpoint inhibitors. Accurate characterization of molecular subtypes, based on gene mutational and expression profiling analyses, will be certainly helpful for individualized treatment and targeted therapy of breast cancer patients, especially for those subtypes with adverse outcome.

## Introduction

Breast cancer is a global public health concern with a high incidence among women, leading to substantial morbidity and mortality [1, 2]. The treatment approach for breast cancer takes into account various factors such as cancer stage and biology, and patient preferences and tolerance. Surgery is the primary treatment option, complemented by radiation, chemotherapy, endocrine therapy, targeted therapy, and immunotherapy [3, 4]. It is essential to recognize that breast cancer exhibits significant heterogeneity in terms of its morphological and molecular characteristics [5, 6]. Gaining a comprehensive understanding of the distinct features associated with breast cancer is vital for accurate diagnosis, optimal treatment selection, and prognostic predictions for patients.

Breast cancer arises in the epithelium of the ducts (85%) or lobules (15%) in the glandular tissue of the breast. According to the PAM50 signature, breast cancer has been classified into five molecular subtypes: Luminal A [estrogen receptor (ER)^+^ and progesterone receptor (PR)^+^, HER2^-^, low levels of Ki-67], Luminal B [ER^+^, HER2^-^, and either Ki-67 high or PR low], Luminal B-like (ER^+^, HER2^+^, any Ki-67, and any PR, HER2-enriched [ER^-^, PR^-^ and HER2^+^], triple negative (TNBC) or basal-like [ER^−^, PR^-^, and HER2^-^] [7, 8]. Heterogeneity of breast cancer makes its treatment complicated, involving a combination of different modalities including surgery, radiotherapy, chemotherapy, hormonal therapy, or targeted biological therapies [9]. Multiple genetic aberrations are present in breast cancer patients [10, 11], ranging from p53 [12–15], components of the apoptotic machinery [16] and of the ubiquitin proteasome system [17–19], transcriptional modifiers [20–22], to hypoxic or metabolic regulators [23–26]. These factors determine cancer progression [27–29] and affect therapeutic responses [30, 31]. Moreover, further analyzes of gene expression or mutational status might reveal additional tumor heterogeneity among subtypes and probably help to develop new personalized treatment strategies for individual patients.

Luminal B breast cancer typically affects women who are post-menopausal but can also occur in younger women; these tumors are more aggressive than subtype A ones. They usually display higher grades and have worse prognosis because of high expression of proliferation-related genes. They also benefit from hormone therapy along with chemotherapy [32]. Tumors with a high mutational load, for instance because of DNA mismatch repair deficiency, are generally highly responsive to immune checkpoint blockade [33]. Luminal-type breast cancers are normally less immunogenic than TNBCs because of a lower rate of tumor specific mutations and subsequent neoantigen load. They are therefore less frequently characterized by the presence of tumor-infiltrating lymphocytes that are often associated with favorable prognosis in TNBC and HER2^+^ breast cancers.

In this case report, we present a detailed analysis of a luminal B breast cancer case, with a high proliferation rate. Genome-wide mutational and global gene expression analyses were performed to characterize the molecular profile of this neoplasia. Microsatellite instability, cancer mutational signatures, and the tumor mutational burden were also evaluated. The aim of this case report is to provide insights into the distinct molecular characteristics of breast cancer and their potential clinical significance. By providing a comprehensive multi-omics analysis of this breast cancer patient, we have proposed potential prognostic indicators for the patient as well as avenues for therapeutic interventions. Ultimately, this knowledge will contribute to the continuous endeavors in combating breast cancer and enhancing the well-being of those affected by the disease.

## Case presentation

### Case narration

**Fig. 1:**
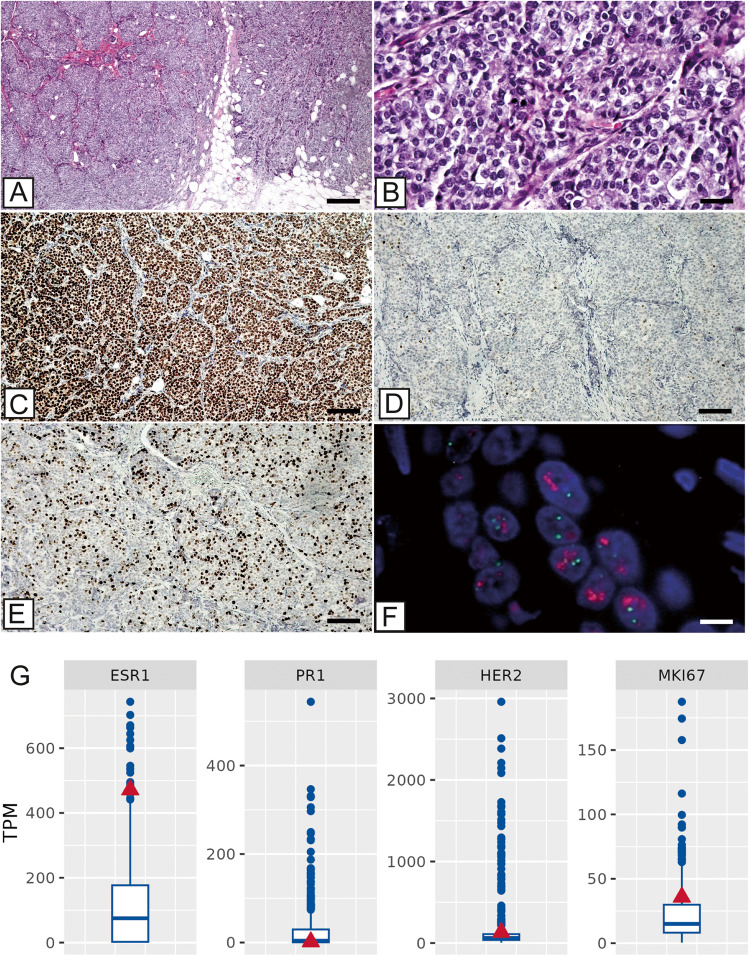
Histopathological and molecular characterization of the tumor. **A** Haematoxylin and eosin slide shows a ductal infiltrating breast carcinoma (G3) (scale bar represents 200 µm). **B** High magnification of panel A shows high cellular pleomorphism, absence of tubules and numerous mitoses (scale bar represents 20 µm). **C** Estrogen receptor expression in more than 95% of breast cancer cells (scale bar represents 200 µm). **D** Progesterone receptor expression in about 1% of breast cancer cells (scale bar represents 200 µm). **E** Image displays high proliferation index (Ki67 > 35%) (scale bar represents 200 µm). **F** In situ hybridization analysis of HER2 status demonstrates the amplification of HER2 gene (ErbB2/CEN17 ratio >2) (scale bar represents 10 µm). **G** Expression mRNA levels (TPM) of estrogen receptor 1 (ESR1), progesterone receptor (PR1), ERBB2 receptor tyrosine kinase 2 (HER2) and proliferation marker KI-67 (MKI67) for the patient (red triangle) and the clinical cohort (blue boxplot).

A female, 47-year-old asymptomatic patient with a BMI of 25.7, non-smoker, non-vegetarian and normal defecation was diagnosed with breast cancer (Table 1). Her anamnesis included menarche at 10 years old and menopause at 40 years old because of bilateral oophorectomy. She had one pregnancy at the age of 30 and did not breastfeed. There is no history of hormone use before menopause. The histo-pathological diagnosis was breast invasive ductal carcinoma NST, tumor grading G3 (Fig. 1A, B). The TNM staging was pT2 pN0 (sn). The patient did not receive neoadjuvant therapy and underwent complete radicality. The patient was also diagnosed with autoimmune thyroiditis.

**Table 1:** Clinical data.

Parameters	Description
Gender	female
Age at case start	47
General condition	Grade 0 - Asymptomatic
BMI	25.7
Vegetarian	no
Meat consumption	4 times per week
Defecation	normal
Smoker	no
Menarche	10 age of year
Menopause	yes
Menopause at	40 age of year
Reason	bilateral oophorectomy
Pregnancies	*n* = 1, at age 36
Breastfeeding	no
Hormone pre menopause	no
Previous disease	Autoimmune thyroiditis from / to:~/04.2021
Tumor type	breast primary tumor
Histological type	ductal carcinoma
Subtype	Luminal B (ER + PR + HER2-)
TNM	pT2pN0 (sn)
Grading	G3
Dignity	malignant
Ki-67	35
Treatment	Total radicality
Neoadjuvant therapy	no

The analysis of the prognostic and predictive biomarkers by immunohistochemistry (IHC) and fluorescence in situ hybridization (FISH) enabled the classification of the breast cancer as luminal B (Fig. 1C–F). In fact, the tumor was found to be ER^+^ ( > 95%), PR^+^ (1%) and HER2 score 2 at IHC. FISH analysis demonstrated the amplification status of HER2 (ErbB2/CEN17 ratio >2 FISH). Proliferation indices evaluated in terms of percentage of Ki67 positive cancer cells were high (Ki-67 > 35%). Of note, ER1 and HER2 expression assessed by RNA-seq is consistent with IHC analysis. As shown in Fig. 1G, the patient indeed showed upregulation of ER1 and down-regulation of HER2 mRNAs as compared to the clinical cohort (354 ER^+^HER2^-^ cases out of total 580 breast cancer patients). Intrinsic subtyping was carried out by using both the research-based 50-gene prediction analysis of a microarray (PAM50) subtype predictor and Absolute Intrinsic Molecular Subtyping. Overall, the diagnosis of luminal B subtype was consistent with both histopathological and molecular data.

### Genetic mutation analysis

Breast cancers seem to be usually caused by the accumulation of multiple mutations and genetic aberrations that correlate with different treatment strategy and clinical outcome of patients [34–36]. In accordance with the high heterogeneity of breast cancer, the gene mutation pattern also varies between different subtypes, and can be unique for every single patient [5, 37–41]. Luminal/ER-positive subtypes are reported to be the most heterogeneous in terms of mutation spectrum, copy number changes and patient outcomes [8, 42].

**Fig. 2:**
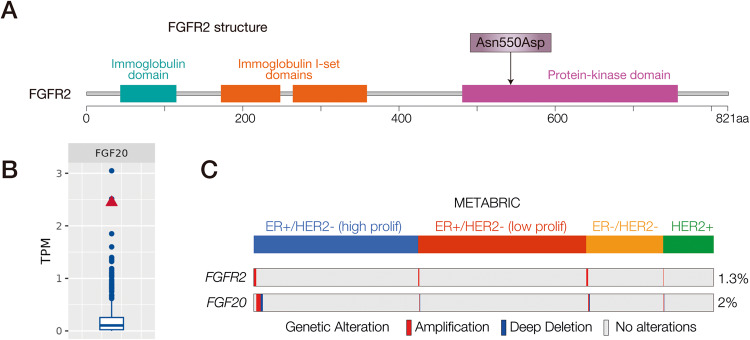
Alterations of the FGFR signaling in the breast cancer patient. **A** Schematic structural features of the FGFR2 protein. Patient’s mutations are indicated by an arrow. Data were obtained from cBioPortal. **B** Expression of *FGF20* in the patient (red triangle) compared to the clinical cohort (RNA-Seq). **C** *FGFR2* and *FGF20* genomic alterations in the METABRIC dataset. Samples were clustered based on their molecular subtype. Data were obtained from cBioPortal.

A comprehensive genomic profiling revealed a high frequency of somatic mutations with single base substitutions in the patient (Table 2). Importantly, among them, there are mutations with available targeted treatment in different phases of clinical trials. It is worth mentioned that we found a new mutation (Asn550Asp) at the *FGFR2* locus, a tyrosine kinase receptor that mediates FGF signaling. Although a different amino-acidic substitution (Asp550Lys) has been identified in breast cancer patients [43], this particular mutation has never been previously described in mammary tumors. The mutation is located within the tyrosine kinase domain (Fig. 2A), potentially leading to aberrant activation of the receptor. Of note, one ligand of FGFR2, *FGF20* was also found to be amplified in the tumor tissue (Table 3). Accordingly, we observed an increase in *FGF20* expression at mRNA level in this patient (Fig. 2B), suggesting that both hyper-activated *FGFR2* and increased levels of its ligand could amplify the FGFR2 signaling. Noteworthy, FGF20 is found amplified and to lesser extent deleted in approximately 2% of breast cancer cases from METABRIC dataset (Fig. 2C).

**Table 2:** Somatic and Germline^a^ mutations.

Symbol	Position	Original AA	Alteration	VAF	Clinical trial phase
DNMT3A	749	Arg	His	60.50%	1–2
MSH6	1024	Arg	Trp	50%	^--^
PIK3CA	1047	His	Arg	42.20%	1–4
ARID1A	372	Gln	Frameshift	40.80%	^--^
SGK1	355	Thr	Met	40.40%	^--^
PARP3	100	Arg	His	40.30%	1–3
PAX5	322	Ala	Frameshift	40%	^--^
POLD1	915	Arg	His	38.60%	1–4
CIC	591	Arg	His	38.50%	^--^
PDGFRA	824	Val	Ile	36.40%	1–2
KMT2A	1350	Arg	Frameshift	36.20%	^--^
CREBBP	1084	Ile	Frameshift	35.80%	^--^
HIF1A	655	Arg	Cys	35.50%	^--^
STAG2	975	Lys	Arg	34.60%	^--^
BAP1	477	Ala	Thr	31.50%	^--^
TSC2	806	Val	Met	31%	^--^
PLCG2	620	Thr	Met	26.40%	^--^
NOTCH3	1665	Ala	Val	25.40%	^--^
DDX41	604	Gly	Arg	22%	^--^
FGFR2	550	Asn	Asp	20.70%	1–2
MSH2^a^	692	Gly	Glu	^--^	1–4
PMS2^a^	415	Val	Met	^--^	1–3

^a^Germline mutations.

**Table 3:** Chromosomal alterations.

Symbol	Variation
ALK	deletion
ASXL2	deletion
CDC73	amplification
CENPA	deletion
DNMT3A	deletion
ELOC	amplification
EML4	deletion
EPCAM	deletion
FGF20	amplification
KAT6A	amplification
LYN	amplification
MSH2	deletion
NBN	amplification
NRG1	amplification
PREX2	amplification
PRKDC	amplification
RAD21	amplification
RFWD2	amplification
RUNX1T1	amplification
SOS1	deletion
SOX17	amplification
UBR5	amplification

**Fig. 3:**
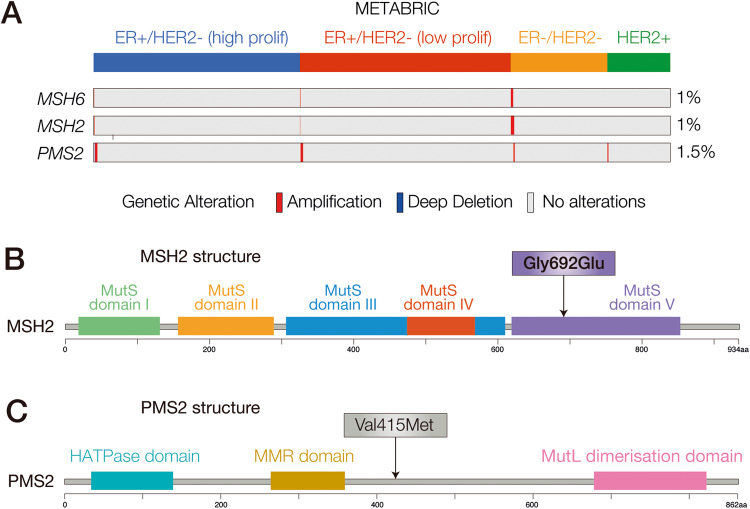
Alterations of the MMR system in the breast cancer patient. **A** *MSH2*, *MSH6* and *PMS2* genomic alterations in the METABRIC dataset. Samples were clustered based on their molecular subtype. Data were obtained from cBioPortal. **B** Schematic structural features of the MSH2 protein. Patient’s mutation is indicated by an arrow. Data were obtained from cBioPortal. **C** Schematic structural representation of the PMS2 protein. Patient’s mutation is indicated by an arrow. Data were obtained from cBioPortal.

**Fig. 4:**
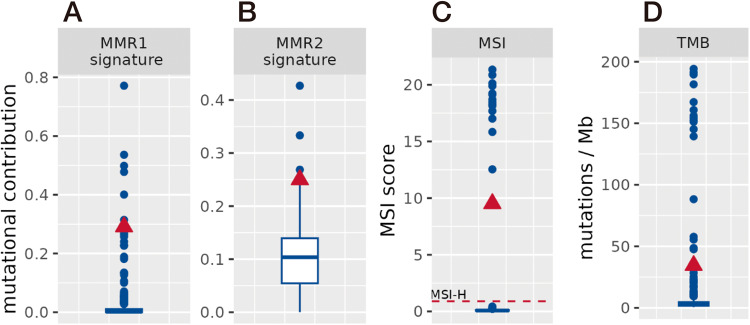
MMR signature, MSI and TMB status of the patient. The patient (red triangle) is compared to the clinical cohort (blue boxplot). **A**, **B** Mutational contribution of MMR related signatures. **C** MSI score (MSI High: score >0.901). The patient is observed as having MSI High status. **D** The patient has a higher TMB as compared to the cohort median (>95. percentile).

We also identified a somatic mutation in the *MSH6* gene (Arg1024Trp) with a high variant allele frequency (VAF) (50%) and two germline mutations in the *MSH2* and *PMS2* genes (Table 2). These three genes, together with *MLH1* constitutes the mismatch repair (MMR) system that is in charge of recognizing and repairing deletions, insertion, and misincorporation of bases during DNA replication and recombination. *MSH2* mutations have been associated with an increased risk of developing several tumors including colon, breast, ovarian, and endometrial cancers [44], however these mutations in breast cancer are quite rare, while amplifications are more frequent with 1% of cases showing overlapping amplification of *MSH6* and *MSH2* and up to 1.5% *PMS2* amplification in other cases from METABRIC database (Fig. 3A). The mutation in the *MSH6* gene has been already reported in colorectal cancer patients in which was associated with medium/high TMB [45]. In this patient, the mutation c.20754G>A (Gly692Glu) of the *MSH2* gene lies within 5th domain (V) (Fig. 3B), is novel and has not been reported previously. We therefore put forward the hypothesis that a mutation within this domain may inhibit MMR [46]. We also found a deletion of the *MSH2* gene (Table 3). Although already described in other tumors as a somatic variant [47], to the best of our knowledge, this *PMS2* germline mutation has never been previously reported in breast cancer patients. The *PMS2* alteration c.1243G>A (Val415Met), is a transition mutation located between the DNA mismatch repair and the dimerization domains (Fig. 3C). Consistent with the observed alterations, mutational signature analysis revealed a strong MMR signature (Fig. 4A, B).

Since the MMR system assists in the maintenance of the genomic fidelity and reduces general gene mutations, its impairment facilitates high frequency somatic mutations and can lead to the insertion or deletion at microsatellites. Coherently, we observed high TMB (34.5) and MSI (9.5) values in this patient as compared to the clinical cohort (Fig. 4C, D). MMR is closely correlated to treatment selection. Chemotherapy and radiation treatment-induced mutagenesis may be accelerated in patients having deficiency in the MMR system. Some novel mutated genes may be driver genes of cancer, which means that MMR inactivation can lead to disease progression and resistance to chemotherapy and radiotherapy. From this point of view, chemotherapy and radiotherapy is probably risky for patients with MMR. On the other hand, previous studies demonstrated that hypermutation and MMR are associated with high tumor-specific neoantigen burden inducing high T cell infiltration that is therefore predictive of a high response to immune checkpoint inhibitors (ICI) [48–51].

**Fig. 5:**
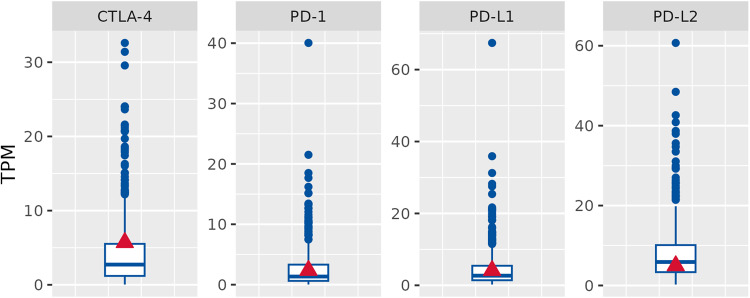
RNA-Seq expression levels of immune checkpoint genes in the patient. The patient (red triangle) is compared to the clinical cohort (blue boxplot).

To find further potential treatment strategies of immune therapy for this case, we assessed the expression of immune checkpoint genes, including cytotoxic T-lymphocyte-associated protein 4 (*CTLA4*), programmed cell death protein 1 (*PD-1*), programmed death-ligand 1 (*PD-L1*), programmed death-ligand 2 (*PD-L2*) by RNA-sequencing. As shown in Fig. 5, *CTLA-4* and PD-1 expression was moderately higher in this patient compared with the median expression in the clinical cohort, while for the other checkpoint genes, the differences were less pronounced.

Furthermore, we also assessed potential risk factors using several risk models. Comprehensive analysis of all these models indicated that this patient is at high risk of recurrence and decreased relapse-free survival. As for therapeutic strategy, the prediction showed increased sensitivity to neoadjuvant paclitaxel plus fluorouracil, adriamycin, and cyclophosphamide chemotherapy, and low risk of tamoxifen resistance.

## Discussion

Breast cancer is a highly heterogeneous disease that can be characterized not only by pathological features, but also according to differences in genetic and molecular characteristics. The heterogeneity of breast cancer has significant implications for the diagnosis, treatment, and prognosis of this disease and personalized treatment for individual patients is recommended. Accurate classification of breast cancer subtypes and further molecular diagnosis are helpful to identify potential targets for personalized therapies and determine the most appropriate treatment options.

This patient was diagnosed with Luminal B subtype breast cancer, predicted with high risk of recurrence and decreased relapse-free survival. By performing global genomic and transcriptomic analyses, we proposed new neoadjuvant treatments for this patient based on identified molecular signatures. The genomic alteration analysis can also help in re-subtyping at molecular level Luminal B breast cancer. At first, the risk model showed low risk of tamoxifen resistance, suggesting that tamoxifen could be probably the basic treatment. We found that *FGFR2* and its ligand *FGF20* are potentially aberrantly activated and over-expressed, due to mutational and genomic alterations respectively. *FGFR2* amplification has also been found in other tumors, such as gastric, esophageal, squamous cell lung, liver, and bladder cancers [52]. Drugs targeting FGFR2 have been already used in clinical trials for treating *FGFR2* amplified tumors, including Bemarituzumab and AZD 4547(*ClinicalTrials.gov*). In this case, both FGFR2 and FGF20 are proposed as potential targets.

In conclusion, a high MSI and TMB pattern and loss of wild-type *MSH2, MSH6 and PMS2* genes, strongly suggest that, in this patient, the mismatch repair defect might have contributed to the development of breast cancer. High frequencies of somatic mutations may result in increased expression of neoantigens, and tumor-specific T-cell reactivity. Hence, considering the high TMB and MSI-H values, and the MMR signature, the patient would be probably sensitive to ICIs. Immune checkpoint detection indicated that ICI targeting CTLA-4 and PD-1 would be another option of neoadjuvant treatment. Fremd and colleagues reported a case of a woman with hormone receptor-positive, HER2 negative metastatic breast cancer, who achieved a durable complete remission after treatment with pembrolizumab [53]. This finding strongly supports the importance of testing the MMR in breast cancer patients and searching for biomarkers to predict the success of the immune therapy. However, whether targeting FGFR2/FGF20 or immune checkpoint genes should be considered only after much more convinced verification of genes expression at protein level. It is worth mentioning that chemotherapy and radiotherapy should be considered carefully because of the possibility of novel mutations of cancer driver genes, which can lead to selection of treatment-resistant cells.

Altogether, we present a comprehensive multi-omics analysis of a case with luminal B breast cancer and propose potential therapeutic strategies based on identified molecular targets.

## Materials and methods

### Collection of samples

Tumor tissues were globally collected using a standardized protocol, minimizing the ischemia time until freezing in liquid nitrogen. To ensure the quality of the samples, all tissues were hematoxilin and eosin stained and subjected to a pathological QC. Samples need to be invasive, have a tumor content of ≥30 % and Necrosis ≤30 %. Normal tissues were processed in parallel and need to be free of tumor and representative regarding the tumor tissue to be included.

Approximately 10 mg tissue were taken for nucleic acid extraction and protein lysate preparation each. To account for tumor heterogeneity, pathological QCs were performed on two sections, before and after taking the analysis material. The tissues stay frozen during the entire process.

### Immunohistochemical analysis

Approximately 1 × 1 × 0.5 cm of tissue was formalin-fixed and paraffin-embedded. Serial sections were used to evaluate prognostic and predictive biomarkers including ER, PR, Ki67, and HER2 through immunohistochemistry. Briefly, sections were stained using the automated Leica Bond IHC platform (Leica Biosystems, Deer Park, IL). After antigen retrieval, 4-μm thick sections were incubated with the following primary monoclonal antibodies: mouse monoclonal anti-ER (clone 6F11; Leica Biosystems), mouse monoclonal anti-PR (clone 16; Leica Biosystems), mouse monoclonal anti-Ki67 (clone MM1; Leica Biosystems) and mouse monoclonal anti-HER2 (clone CB11, Leica Biosystems). Reactions were revealed using BOND-PRIME Polymer DAB Detection System (Leica Biosystems, Deer Park, IL). Immunohistochemistry was evaluated by two blind pathologists.

### Nucleic acid extraction and quality assessment

Frozen tissue slices were mixed with β-mercaptoethanol containing sample buffer and homogenized using the BeadBug system. DNA and RNA were extracted in parallel from the same sample using the Qiagen AllPrep Universal Kit according to the manufacturer’s instructions.

DNA and RNA concentration were quantified using Qubit fluorometer with the Qubit dsDNA BR assay or Qubit RNA BR assay respectively.

DNA and RNA quality were assessed using the Agilent Tapestation with the Agilent Genomic DNA kit or Agilent High-Sensitivity RNA ScreenTape kit respectively. RNAs need to have a RIN ≥ 4 or a DV200 ≥ 60 to be selected for library preparation.

### Library preparation and NGS sequencing

Libraries for whole genome sequencing (WGS) were prepared using the PCR-free KAPA Hyper Prep Kit (Roche). For whole transcriptome sequencing, RNA samples were depleted of the ribosomal RNA using the Ribo Zero Kit (Illumina) and library preparation was performed using the TruSeq Stranded Total RNA Kit (Qiagen). For small RNA sequencing the QIAseq miRNA Kit (Qiagen) was used All library preparation kits were used according to manufacturer’s instructions. Sequencing was performed on a NovaSeq6000 system (Illumina).

For WGS, average coverage for tumor samples was ≥60× and ≥30× for normal samples with a total genomic coverage of ≥95%.

Whole transcriptome sequencing datasets have ≥100 million total reads with less than 20% of ribosomal origin and ≥20 million reads mapping to mRNAs according to Ensembl reference. Ribosomal depletion was performed to remove nuclear rRNA and mt-rRNA.

### NGS data processing

NGS data were aligned against Grch38 genome assembly. Identification and annotation of short genomic variations in normal sample was done using Haplotype Caller (genome analysis toolkit; GATK) [54]. WGS somatic variation were called using a consensus of Mutect2 [55], Strelka [56], Varscan [57], and Somatic Sniper [58]. Structural variations were called using R packages TitanCNA [59] and DellyCNV [60].

RNA-Seq differential expression was based on normalized readcount data (TPM: transcripts per million).

### Bioinformatical analyses

Mutational signatures were calculated using the R package MutationalPatterns [61]. MSI classification was done using R package MSIseq [62]. PAM50 subtyping as well as risk scores were investigated using R package genefu [63]. TMB was calculated as the number of non-synonymous mutations of protein coding genes divided by exome size in Megabases.
